# Cases in Surgery—Abscess in Long Bones

**Published:** 1870-01

**Authors:** H. O. Hitchcock

**Affiliations:** Kalamazoo, Mich.


### Article III. — *Cases in Surgery — Abscess in Long Bones.*

By H. O. Hitchcock, A.M., M.D., Kalamazoo, Mich.

Case I. — Probable abscess of the humerus following a blow; caries of the whole bone; amputation at the shoulder-joint; recovery.

C. S., of Climax, Mich., suffered somewhat during his military service — 1862 to 1865 — from camp diarrhœa, but had pretty fairly recovered from its effects. In February, 1868, while at work drawing wood, he received quite a severe blow about midway of the right arm, not so hard, however, as to fracture the bone or greatly to contuse the flesh.

This was soon followed by severe, deep-seated pain, from the middle of the arm to near the elbow-joint. The pain was dull, heavy, and almost unendurable, and accompanied by considerable swelling, which was poulticed for a number of weeks, after which an abscess was opened near the elbow, which opening never healed. The discharge of large quantities of sanious pus did not wholly relieve the pain which then appeared to be in the bone itself.

The disease has been steadily progressing ever since — new sinuses now and then opening, but never healing.

In the summer of 1869 an attempt was made to remove a suspected sequestrum in the upper half of the bone; but the bone was found to be so extensively carious the attempt was abandoned, and the disease was allowed its natural progress, except as modified by constitutional treatment.

The patient came to my office October 22, when the arm presented the following appearance: Nearly complete anchylosis of the elbow joint, with several sinuses leading down to both condyles of the humerus, which appeared to be carious; several sinuses along the arm leading down to the excavated medullary cavity, with two at the inner aspect of the shoulder joint, with thickening of the bone throughout its whole extent, and great infiltration of the soft parts.

Diagnosis, from history and present appearance, "caries of the whole humerus taking its origin in periostitis or endostitis, or both, with probable abscess in the shaft of the bone."

As the patient's health was greatly suffering from the constant irritation and discharge, and as the whole bone, including its articular ends, appeared to be carious, I advised immediate amputation at the shoulder joint. This operation I performed October 26, 1869, making the oval operation of Baron Larrey.

The operation was quite a difficult and bloody one, on account of the greatly thickened soft parts around the joint, all of the blood vessels being greatly enlarged. The head of the humerus was found to be entirely carious.

The patient, after the operation, rallied very well. He was left in the charge of Dr. Seeley, of Climax Prairie, who informs me that the wound was completely healed and the patient in good health one month after the operation.

Case 2. — Probable abscess of the shaft of the femur after severe injury to the thigh, followed by internal necrosis, and extensive sub-periosteal abscess. Bone trephined. Cavity scraped. Recovery. P. V. W., a large, well-developed man, of 37 years, with no taint of syphilis or scrofula, a builder by occupation, presented himself at my office, Nov. 15, 1859, for examination and advice.

Five or six years previous, while raising the roof of a church, he received a severe raking injury in the right thigh, by the falling of heavy timbers upon its outer aspect. At the same time there was fracture and dislocation of the left ankle. The injury to the right thigh was considered by the surgeon then in attendance only muscular.

But a few weeks after the accident the patient began to suffer very severe pain in the right thigh, deep seated, as if in the bone. This, he says, was considered and treated as a rheumatic pain for three weeks, when, choosing to die rather than be in such agony longer, the patient insisted that his medical attendant should open it. This gave exit to a large quantity of sanious, watery pus, and afforded great relief to the patient. The cut of the knife has since that time remained as a sinus, continuing to discharge pus of the same character.

Six months after the accident the patient resumed, and has since continued, his occupation, though with much pain and great discomfort. While in the upright position, at first, but little oozing took place, but the lower half of the thigh would soon become swollen and painful, " as if," in his own expressive words, "a dog were gnawing it." And when the abscess was nearly filled, every contraction of the muscles of the thigh caused the matter to flow from the sinuses. In hot days, in summer, this discharge was often immense, and at night he would close the labors of the day by evacuating the pus from the lower part of the thigh with his foot elevated. Such had been his life for five years.

On making, December 15, 1859, an incision ten inches in length between the biceps and vastus externus muscles, commencing one and a half inches above the knee-joint, the bone was found bare of its periosteum almost the entire length of the incision, and for more than one-half of the circumference of the bone. The surface of the bone was granular and somewhat crumbly. In the upper half of the middle third the femur seemed decidedly and somewhat abruptly enlarged, even to nearly twice its normal size. At this point of enlargement was found a round, as if smoothly bored, hole, about a quarter of an inch in diameter, leading to the medullary cavity of the bone. This hole was filled with a jelly-like substance, such as also covered the bone where denuded of its periosteum. The bone was, at this point, trephined, and a sequestrum, one inch long, by one-third of an inch wide, and one-sixth of an inch thick, was removed from the medullary cavity. The cavity was thoroughly cleansed by scraping away all granular and crumbly bone, as was also the denuded exterior of the bone, and in a few weeks the cure was complete.

About the time case No. 1 came under my notice, my eye fell upon the article of Dr. Geo. C. Blackman, in the October No. of the *American Journal of the Medical Sciences*, "*on certain points connected with the pathology and treatment of abscess in bone;*" and it seemed to me that the two cases above related, while contrasting with each other in many respects, are both typical cases to illustrate the history of abscess in bone when neglected.

Dr. Markoe, in a paper in the *New York Journal of Medicine*, May, 1858, on "Chronic Sinuous Abscess of Bone," as quoted by Dr. Blackman, describes a class of cases very like the above: "distressing, tedious, intractable;" "cases in which the inflammation of the bone begins as an acute attack, passing rapidly into suppuration, and in which the abscess, thus rapidly formed, finds its way early to the surface, through the compact external shell of the bone, and is discharged, to the temporary relief of the sufferings of the patient, though it may be not greatly to his advancement towards a cure. * * * The opening not being free, and probably not being direct, accumulations of matter take place within the cavity, and new inflammations and suppurations are excited in the bone substance surrounding the original focus of disease; * * * and the bone is left perforated in all directions, by two, three, four or more, sinuses, generally all communicating with one another, and with a central excavation or chamber which marks the position of the original abscess."

The above quotation is a complete description of the history of the disease in the first case here reported.

The second case is in somewhat of contrast to this, for several reasons. The sinus in the bone was a direct and short one, and had the sinus in the soft parts corresponded exactly to that in the bone, the inconvenience to the patient would have been much less. But as the sinus in the soft parts was at some distance from that in the bone, the discharged matter dissected up the periosteum and made a large sub-periosteal abscess. Besides, in the second case, the bone around the sinus and abscess appeared to have become enlarged and eburnated, while that in the first case became carious.

This difference might have been owing to the difference in constitution and condition of the two patients at the time of the injury.

Mr. Hey, of Leeds, in his *Practical Observations in Surgery*, describes a case very similar to case 2. "I found," he says, "the periosteum diseased and thickened. The surface of the tibia was rough as far as the matter had covered it; and in the centre of the rough part there was a hole equal in bore to a goose's quill, which penetrated the bone transversely about a quarter of an inch." Mr. Hey, in this case, sawed out a wedge-shaped piece of bone, two inches in length, from the tibia, and thoroughly scraped and gouged out the diseased surface of the cavity, and his patient made a complete recovery.

In this case the pus seems to have been six weeks in coming to the surface, but was not opened till one year after. He says: "The pain was so great during this operation of nature, that, my patient assured me, and that immediately after the removal of the carious part of the bone," (the operation was done before the days of chloroform) "that she had suffered more pain during the whole of the six weeks above mentioned, unless she was asleep, than I had caused during the operation necessary for removing the unsound bone." "It is surprising that such a perforation should have been made through so firm a part of the bone without any extensive caries in the lamella." "The perforation appeared as if it had been made with a gimlet."

Dr. Nathan Smith, in a paper on necrosis, in his *Medical and Surgical Memoirs*, edited by his son, Dr. Nathan R. Smith, referring to the acute inflammation which precedes the death of the bone, remarks: "When the shafts of the long bones are the seats of the disease, about the same time that matter is deposited beneath the external periosteum, there is formed a corresponding collection between the internal surface of the bone and the membrane surrounding the medullary surface, so that there exist two collections of matter, bathing the opposite sides of the walls of the bone. This fact, which I deem of great importance, as being essential to the correct treatment of the disease, I have ascertained, in repeated instances, by the operation which I have performed for its relief, namely, the trepanning the bone."

Dr. Blackman quotes several cases by Dr. Benjamin B. Simons, of South Carolina, in which he trephined bones for abscess, in various stages of the disease, in seven cases, all of which completely recovered.

In the first case reported in this article, had the humerus been trephined soon after the accession of the severe pain which followed the blow, in all probability the patient would have recovered with a sound and useful arm. In the second case, had the same operation been performed, a like result would probably have followed, and the patient been spared five or six years of great pain and discomfort.

These two cases teach us the great importance of distinguishing the symptoms which indicate abscess of the bones, and of prompt action for their relief. Here is a fine field for true conservative surgery, not to give the management of disease up to nature, but promptly, and in good time, to interpose a minor operation, so as to arrest the natural progress of disease, and to prevent the necessity of a more formidable operation, and the loss of limb and of life.

